# Comparison between 6 weeks of static stretching and resistance training programs on passive and active properties of plantar flexors. a randomized controlled trial

**DOI:** 10.3389/fphys.2025.1555253

**Published:** 2025-10-15

**Authors:** Yuta Murakami, Andreas Konrad, Kazuki Kasahara, Riku Yoshida, Konstantin Warneke, David G. Behm, Masatoshi Nakamura

**Affiliations:** ^1^ Department of Rehabilitation, Yamagata University Hospital, Yamagata, Japan; ^2^ Institute of Human Movement Science, Sport and Health, University of Graz, Graz, Austria; ^3^ Sanyudo Hospital, Yonezawa-shi, Yamagata, Japan; ^4^ Department of Rehabilitation Medicine, Maniwa Orthopedics Clinic, Niigata, Niigata, Japan; ^5^ Institute of Psychology, Leuphana University Lüneburg, Lüneburg, Germany; ^6^ School of Human Kinetics and Recreation, Memorial University of Newfoundland, St. John’s, NL, Canada; ^7^ Faculty of Rehabilitation Sciences, Nishi Kyushu University, Kanzaki, Japan

**Keywords:** range of motion, passive torque, stretch tolerance, passive stiffness, muscle strength, muscle thickness, pennation angle, calf raise exercise

## Abstract

**Introduction:**

Resistance training (RT) and static stretching (SS) are both exercises that increase range of motion (ROM), muscle strength, and muscle mass. This study aimed to compare the effects of SS and RT and examine factors related to the increase in ROM, muscle strength, and morphology.

**Methods:**

Thirty-six healthy untrained male adults (age: 21.7 ± 1.2 years) were allocated to SS, RT, or control (no intervention) groups for a 6-week intervention program. Dorsiflexion (DF) ROM, passive torque at DF ROM, passive stiffness, maximal voluntary isometric contraction (MVC-ISO), MVC concentric (MVC-CON) and MVC eccentric (MVC-ECC) torques, and muscle thickness of plantar flexors were measured before and after the intervention.

**Results and discussion:**

Both SS and RT groups increased DF ROM (SS: p < 0.01, d = 0.65, RT: p = 0.038, d = 0.37) and passive torque at DF ROM (SS: p = 0.027, d = 0.64, RT: p < 0.01, d = 0.41) with similar small to moderate effect size magnitudes, while only the SS group experienced a significant, small magnitude decrease in passive stiffness (p = 0.025, d = −0.32). MVC-ISO, MVC-CON at 30°/s, and MVC-ECC torques at 30°/s showed small to large magnitude, significant increases in muscle strength (MVC-ISO at 30° plantarflexion: p < 0.01, d = 1.00, MVC-ISO at neutral position: p < 0.01, d = 0.43, MVC-ISO at 15° dorsiflexion: p < 0.01, d = 0.43, MVC-CON at 30°/s: p < 0.01, d = 0.38, MVC-ECC at 30°/s: p = 0.023, d = 0.48), whereas muscle thickness at medial and lateral gastrocnemius muscle (p < 0.001, d = 0.56 and p < 0.01, d = 0.66, respectively) exhibited significant, small magnitude increases only in the RT group. A significant positive correlation was found between the change in DF ROM and the change in passive torque at DF ROM in both SS (p < 0.001, r = 0.863) and RT (p < 0.001, r_s_ = 0.825) groups, but no significant correlation was found between the change in DF ROM and passive stiffness. SS and RT increased ROM similarly, and both ROM increases may be due to changes in stretch tolerance. If increasing ROM and muscle strength is the goal, RT should be selected; conversely, if changes in ROM and passive stiffness are the goal, SS should be selected.

## Introduction

Resistance training (RT) is well known for its positive influence on muscle strength and muscle mass (Schoenfeld et al., 2017). Recently, it has been reported that if RT is performed through the full available range of motion (ROM), it can be effective in increasing the ROM similar to static stretching (SS) training (Alizadeh et al., 2023; Afonso et al., 2021). Previous studies suggest that a decreased ROM could be a contributing factor to sports-related injuries (Knapik et al., 1991; Timmins et al., 2016). Thus, RT could help prevent sports-related injuries by increasing muscle strength and ROM. On the other hand, stretching has historically been used ubiquitously to increase ROM (Konrad et al., 2023). Historically, although RT was the primary means to increase muscle strength and mass, recent research reported that SS can also increase muscle strength and muscle mass (Arntz et al., 2023; Panidi et al., 2023; Thomas et al., 2022). Moreover, performed with sufficient volume, static stretching (1 h daily for 7 days per week) was equally effective in increasing muscle strength, mass, and flexibility compared to 5 sets of 12 repetitions, 3 days per week RT in the plantar flexors (Warneke et al., 2023a) and pectorals (Wohlann et al., 2024).

While stretch-induced ROM improvements are often attributed to decreases in muscle stiffness (Moritani and deVries, 1979) and increased stretch or pain tolerance (Konrad et al., 2023; Nakamura et al., 2020), the mechanisms underlying stretch-induced hypertrophy and strength increases remain poorly investigated (Warneke et al., 2023a). In contrast, it is generally accepted that the mechanism of increased muscle strength can be attributed to both muscle morphological and neural adaptations (Moritani and deVries, 1979). The limited research on ROM increases with RT calls for further investigations to clarify their possible shared functional and morphological mechanisms. Also, several systematic reviews have investigated the chronic effect of SS and/or RT on ROM (Alizadeh et al., 2023; Afonso et al., 2021; Konrad et al., 2023), whereas few studies have comprehensively compared the changes in passive muscle properties, muscle strength, muscle architecture, and stretch tolerance after SS and RT. Moreover, the underlying mechanisms contributing to ROM improvements remain unclear, particularly whether they are driven primarily by changes in tissue stiffness, stretch tolerance, or structural adaptations, and whether these mechanisms differ between SS and RT. To assess differences in the training effects obtained by SS and RT, this study aimed to compare the effects of 6 weeks of SS and RT on muscle passive properties, strength, and architecture, as well as to examine factors related to the increase in ROM. These findings may provide important insights when determining whether SS or RT should be chosen in clinical practice. The primary goal of this study was to investigate possible differences between SS and RT. Previous studies showed that SS and RT within a time frame of 6 weeks showed changes in the parameters assessed (Warneke et al., 2023a; Nakamura et al., 2020). Hence, we decided to perform 6 weeks intervention period in this study.

Due to similarities in the outcome regarding ROM, strength, and muscle mass, it was hypothesized that the 6 weeks of SS and RT interventions would similarly induce improvements in passive stiffness and pain tolerance between SS and RT compared to a passive control group. We hypothesized that muscle strength and thickness would increase similarly with SS and RT.

## Methods

### Experimental design

**FIGURE 1:**
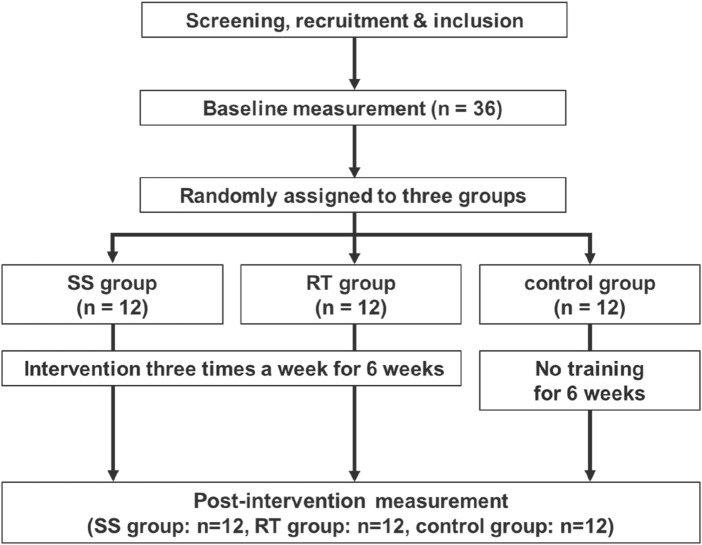
Experimental flow chart.

A randomized controlled trial was used to investigate the chronic effects (6 weeks) of SS and RT intervention, compared to a passive control. Thus, ankle dorsiflexion (DF) ROM, passive torque at DF ROM, musculotendinous (MTU) passive stiffness, muscle strength [maximal voluntary isometric (MVC-ISO), concentric (MVC-CON), and eccentric (MVC-ECC) contraction torques], and muscle architecture (muscle thickness and pennation angle) of plantar flexors in the dominant leg (preferred to kick a ball) (Nakamura et al., 2021a) were investigated before (PRE) and after (POST) a 6 weeks training program. The primary outcome measure is DF ROM. The measurements started with muscle architecture via ultrasound scanning, followed by DF ROM, MVC-ISO, MVC-CON, and MVC-ECC torque measurements. To prevent any effect from the last exercise session, all POST measurements were repeated at least 72 h after the final intervention program session. Furthermore, to eliminate the influence of measurement time, these measurements were conducted during the same time period for both PRE and POST. The control group did not receive an intervention throughout the study (Figure 1). All participants were instructed to refrain from stretching, therapeutic massage, and RT outside of the study during their participation.

#### Participants

The sample size calculation (primary outcome: ROM) for a two-way repeated-measures analysis of variance (ANOVA) [effect size = 0.4 (large), α error = 0.05, and power = 0.80] was performed via G* power 3.1 software (Heinrich Heine University, Düsseldorf, Germany), based on a previous study (Nakamura et al., 2020), and the required number of participants was 36 participants in this study. Consequently, thirty-six (36) healthy untrained male adults participated in this study (age: 21.7 ± 1.2 years, height: 172.0 ± 5.0 cm, and body mass: 61.8 ± 6.3 kg). Inclusion criteria were as follows: no regular stretching and resistance training within the past 6 months, no neuromuscular disease, and no history of orthopedic disease (Nakamura et al., 2022). All participants provided written informed consent after being fully informed of the study procedures and purposes. Participants were randomly assigned to 3 groups: SS (n = 12, age, 22.1 ± 0.8 years; 172.0 ± 5.2 cm; mass 61.7 ± 5.7 kg), RT (n = 12, age, 22.0 ± 0.9 years; 170.1 ± 4.9 cm; mass 61.0 ± 8.0 kg), and control (n = 12, age, 21.0 ± 1.4 years; 174.0 ± 4.0 cm; mass 62.7 ± 4.6 kg) group using a computerized random number function in Microsoft Excel (Microsoft Corp., Washington, WA, United States). Due to the nature of the intervention, complete blinding of the participants and researchers was not feasible, and the researcher responsible for randomization was aware of the study objectives. There were no significant differences in age, height, or mass between groups. This study was conducted following the Declaration of Helsinki and was approved by the Niigata University of Health and Welfare, Japan (ethics approval number: #19063). The study was registered with the University Hospital Medical Information Network Clinical Trials Registry (UMIN: R000067516).

#### Outcome assessment

##### DF ROM, passive torque at DF ROM, and passive stiffness

The participant was secured in a seated position on the chair of an isokinetic dynamometer (Biodex System 3.0, Biodex Medical Systems Inc., Shirley, NY, United States), with a knee angle of 0° (i.e., anatomical position). The trunk and pelvis of the participant were fixed with a belt while the participant was reclined (with the hip angle at 70°) to prevent tension at the back of the knee (Nakamura et al., 2020). Thereafter, the footplate of the dynamometer was passively and isokinetically dorsiflexed at a speed of 5°/s from the 30° plantar flexion to the dorsiflexion angle just before participants started to feel discomfort or pain (Akagi and Takahashi, 2013). After two familiarization trials, the participant stopped the dynamometer by activating a safety trigger when they started to feel discomfort or pain. The angle was defined as the DF ROM (°) (Nakamura et al., 2021a; Nakamura et al., 2021b). The measurement was performed twice, and the maximum value was used for analysis. In addition, passive plantar flexor resistive torque was measured during the DF ROM measurement, and passive torque at DF ROM (Nm) was defined as the passive plantar flexion torque at the time maximum DF ROM was measured (Fukaya et al., 2020). Passive torque at DF ROM was an indicator of the stretch tolerance (Weppler and Magnusson, 2010; Mizuno et al., 2013). Furthermore, to confirm that there was no voluntary contraction of the plantar flexor muscles, electromyography (FA-DL-720-140; 4Assist, Tokyo, Japan) was used. Consequently, surface electrodes (Blue Sensor N, Ambu A/S, Ballerup, Denmark) were applied to the medial gastrocnemius muscle (Nakamura et al., 2013). The passive stiffness (Nm/°) was calculated from the passive torque at DF ROM at a certain angle, based on a previous study (Fukaya et al., 2020). For the calculation, the slope of torque to angle from the last 50% of the smallest DF ROM to the maximum angle (100%) among the DF ROMs was measured four times, both PRE and POST. The measurements were repeated twice, and the minimum of these measurements was used in the analyses.

##### Maximal voluntary isometric contraction torque

MVC-ISO torque was measured in three positions, which were the same positions as those in the DF ROM assessment: 1) at the ankle joint at 30° plantar flexion, 2) in the neutral position, and 3) at 15° dorsiflexion (Sato et al., 2020a). MVC-ISOs were performed for 2 sets with 3 s, with a 60-s rest between each set. Throughout the measurement, participants were verbally encouraged during muscle contraction to promote maximal efforts.

##### Maximal voluntary concentric and eccentric contraction torques

ROM was from 10° of dorsiflexion to 20° of plantar flexion, with an angular velocity of 30°/s and 120°/s for MVC-CON (Yahata et al., 2021) and 30°/s for MVC-ECC (Sato et al., 2020b; Borges et al., 2017). In addition, we applied concentric and eccentric contraction protocols three times in each sequence. Participants were verbally encouraged during muscle contraction throughout the measurement to promote maximal efforts. We measured the maximum torque during both concentric and eccentric contractions were obtained between 10° of dorsiflexion and 20° of plantar flexion.

##### Muscle thickness and pennation angle

Participants were instructed to lie relaxed on the treatment table in the prone position with hip and knee angle at 0° with ankle angle at slack position (Yahata et al., 2021). B-mode ultrasonography (LOGIQ e V2; GE Healthcare Japan, Tokyo, Japan) with an 8 MHz linear array probe was used to evaluate the muscle thickness and pennation angle of the medial and lateral gastrocnemius muscle (MG and LG, respectively). Longitudinal ultrasound images were obtained for the MG and LG at 30% of the lower leg length, measured from the popliteal crease to the lateral malleolus near the point of the maximal cross-sectional area of the lower leg (Akagi and Takahashi, 2013; Yahata et al., 2021; Nakamura et al., 2014). Additionally, a longitudinal ultrasound image of the soleus was obtained at 50% of the lower leg length (Yahata et al., 2021; Kubo et al., 2014; Kubo et al., 2017). All measurements were performed by the same physical therapist, who had over 3 years of experience practicing ultrasound assessment. Image processing software determined muscle thickness and pennation angle (ImageJ, National Institutes of Health, Bethesda, MD, USA). Muscle thickness was determined as the mean of the distances between the deep and superficial aponeuroses measured at both ends of each image (Yahata et al., 2021; Blazevich et al., 2006; Ema et al., 2013). Additionally, pennation angle was determined as the mean of the three fascicles at the angle between fascicle and deep aponeurosis (Sato et al., 2020a; Yahata et al., 2021). MG, LG, and soleus images were measured in triplicate, and the average of the three measurements obtained for both muscle thickness and pennation angle was used for further analysis.

### Interventions

#### Static stretching program

**FIGURE 2:**
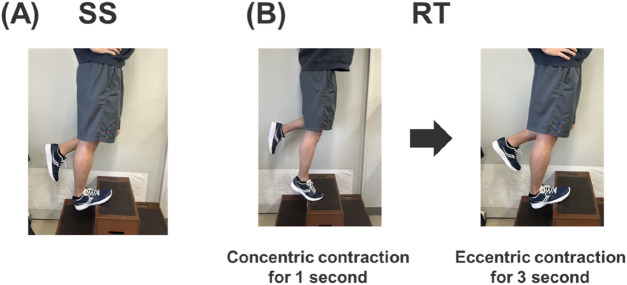
Static stretching SS, **(A)** and resistance training RT, **(B)** intervention set-up.

Participants in the SS group performed a one-legged standing static stretching program, consisting of 3 sets of 40 s, three times per week for 6 weeks under the supervision of the research team. The participant was placed in a one-legged standing position with one leg on a platform and the opposite arm placed against the wall beside the body for balance (Figure 2A). Stretching intensity was defined as the greatest tolerated dorsiflexion angle without pain or discomfort (Sato et al., 2020a; Yahata et al., 2021). By moving the trunk forward, each subject was instructed to maintain a defined intensity. Between every training session, a rest of at least 24–48 h was ensured (Nakamura et al., 2020; Nakamura et al., 2021a).

#### Resistance training program

Participants in the RT group performed the plantar flexor RT 3 times per week for 6 weeks. The calf raise RT program was performed on the dominant leg with an extended knee joint under the supervision of the research team. The exercise ROM involved movement through full plantar flexion to full DF position. Based on the documented effectiveness of eccentric contractions for increasing ROM (Kay et al., 2023), the calf raise exercise consisted of a 1-s concentric followed by 3-s eccentric contractions (Figure 2B). Three sets of 10 repetitions with body weight were performed, with a 60-s rest between sets (Murakami et al., 2024). Between each training session, a 24–48 h rest period was ensured.

#### Control group

Participants in the control group did not receive any intervention, and PRE and POST measurements were performed at 6 weeks intervals. Participants were instructed not to perform stretching or RT for 6 weeks.

#### Test-retest reliability of the measurements

Test-retest reliability was assessed using the coefficient of variation (CV) and the intraclass correlation coefficient (ICC) in 7 participants (age: 20.9 ± 1.0 years, height: 176.1 ± 3.9 cm, and body mass: 64.6 ± 2.8 kg) on different days without any intervention. These participants were different from those included in this study. The CV and ICC of the measurements are shown in Table 1. The ICC ranged from 0.810 to 0.992, and the CV ranged from 1.5% to 13.1%.

**TABLE 1:** Reliability for the test-retest values by intraclass correlation coefficient (ICC) and coefficient variation (CV).

	ICC (95%-CI)	CV (mean ± SD)
DF ROM (°)	0.897 (0.565–0.981)	13.1 ± 12.5
Passive torque at DF ROM (Nm)	0.786 (0.241–0.959)	6.2 ± 2.9
Passive stiffness (Nm/°)	0.992 (0.958–0.999)	1.5 ± 1.1
MVC-ISO at 30° plantarflexion (Nm)	0.966 (0.836–0.994)	5.9 ± 4.1
MVC-ISO at neutral position (Nm)	0.972 (0.866–0.995)	4.3 ± 2.2
MVC-ISO at 15° dorsiflexion (Nm)	0.957 (0.796–0.992)	6.4 ± 3.0
MVC-CON at 30°/s (Nm)	0.941 (0.733–0.989)	4.8 ± 3.3
MVC-CON at 120°/s (Nm)	0.810 (0.301–0.964)	7.6 ± 4.0
MVC-ECC at 30°/s (Nm)	0.925 (0.669–0.986)	6.3 ± 6.7
Muscle thickness at MG (mm)	0.979 (0.896–0.996)	1.8 ± 1.2
Muscle thickness at LG (mm)	0.945 (0.748–0.990)	4.6 ± 5.5
Muscle thickness at soleus (mm)	0.755 (0.165–0.952)	2.3 ± 1.3
Pennation angle at MG (°)	0.754 (0.164–0.952)	6.0 ± 2.6
Pennation angle at LG (°)	0.739 (0.132–0.949)	2.8 ± 4.0

### Statistical analysis

Statistical analysis was performed using SPSS (version 28.0; SPSS Japan Inc., Tokyo, Japan). The Shapiro-Wilk test was used to confirm the normality of the data. The results showed that the rate of change in DF ROM at RT did not follow normality, but the other measures did. The one-way ANOVA was used for each variable to reveal that the PRE values did not differ among the groups (Table 2). All outcome variables were examined for interaction effects using 2 × 3 two-way ANOVA of variance (time [PRE vs. POST] and group [SS vs. RT vs. Control]), and main effects were examined when no significant interaction effects were found. Effect sizes are presented as partial eta-squared values (ƞ_p_^2^) and categorized as either small effect (ƞ_p_^2^ < 0.01), medium effect (ƞ_p_^2^ = 0.02–0.14), or large effect (ƞ_p_^2^ > 0.14) (Cohen, 1988). If a significant interaction effect was detected, a *post hoc* test was conducted using a paired t-test with Bonferroni correction on each group to determine differences between PRE and POST values. Additionally, we calculated Cohen’s d effect size as differences in the mean value divided by the pooled standard deviation between PRE and POST in each group and classified as either trivial (d < 0.2), small (d < 0.5), medium (d = 0.5–0.8), or large effect (d > 0.8) (Cohen, 1988). When significant changes were observed in the two groups in the *post hoc* test, the respective rates of change were compared using an unpaired t-test when normality was followed, and the Mann-Whitney U test when normality was not followed.

**TABLE 2:** Comparison of pre-values in static stretching (SS), resistance training (RT), and Control groups.

	SS	RT	Control	One-way ANOVA
DF ROM (°)	16.0 ± 6.3	17.0 ± 8.5	17.5 ± 7.0	p = 0.889
Passive torque at DF ROM (Nm)	26.7 ± 6.8	31.0 ± 10.2	27.8 ± 8.0	p = 0.466
Passive stiffness (Nm/°)	0.61 ± 0.16	0.71 ± 0.20	0.68 ± 0.19	p = 0.443
MVC-ISO at 30° plantarflexion (Nm)	48.9 ± 17.3	49.6 ± 11.4	47.3 ± 20.5	p = 0.951
MVC-ISO at neutral position (Nm)	136.6 ± 38.5	130.3 ± 34.9	118.4 ± 47.4	p = 0.572
MVC-ISO at 15° dorsiflexion (Nm)	180.7 ± 53.2	163.6 ± 45.0	153.0 ± 63.3	p = 0.491
MVC-CON at 30°/s (Nm)	117.9 ± 21.0	112.2 ± 18.9	97.9 ± 33.5	p = 0.177
MVC-CON at 120°/s (Nm)	70.7 ± 12.7	69.7 ± 14.5	62.4 ± 21.0	p = 0.444
MVC-ECC at 30°/s (Nm)	182.5 ± 44.2	171.6 ± 42.6	159.4 ± 64.7	p = 0.580
Muscle thickness at MG (mm)	18.7 ± 2.8	17.9 ± 1.6	17.3 ± 2.9	p = 0.421
Muscle thickness at LG (mm)	15.1 ± 1.8	13.4 ± 2.1	14.2 ± 1.8	p = 0.149
Muscle thickness at soleus (mm)	16.9 ± 2.0	17.1 ± 2.8	16.8 ± 2.1	p = 0.944
Pennation angle at MG (°)	20.7 ± 3.3	21.3 ± 3.8	21.8 ± 5.7	p = 0.842
Pennation angle at LG (°)	13.4 ± 2.0	12.6 ± 2.1	13.6 ± 2.1	p = 0.462

In addition, the relationship between the change in DF ROM and the change in passive torque at DF ROM and the passive stiffness in the SS and RT groups was examined. Since only the change in DF ROM in the RT group did not follow normality, we examined it using the Pearson product-moment correlation coefficient in the SS group and Spearman’s rank correlation coefficient in the RT group. A *p* value of <0.05 indicates statistical significance.

## Results

### DF ROM, passive torque at DF ROM, and passive stiffness

**FIGURE 3:**
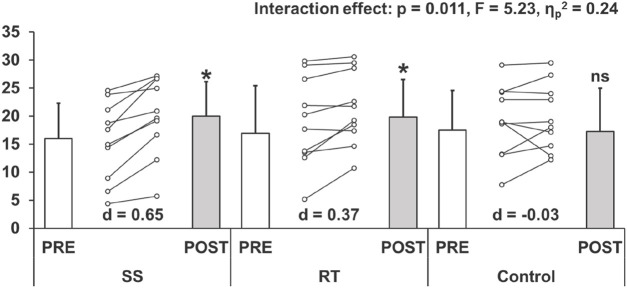
Changes (mean ± SD) in dorsiflexion range of motion before (PRE) and after (POST) the intervention (ststaic stretching: SS, resistance training: RT, and control groups). Cohen’s d effect size (ES) is also included for the significant changes. *: significant (p < 0.05) difference from the PRE value, ns: no significant difference from the PRE value.

Figure 3 shows the results for the DF ROM in both PRE and POST in each group. DF ROM showed a significant interaction effect. The *post hoc* test showed a significant moderate and small magnitude increase after the intervention in the SS (*p* = 0.001, *d* = 0.65) and RT (*p* = 0.038, *d* = 0.37) groups, respectively, with no significant change in the control group (*p* = 1.000, *d* = −0.03). However, the extent of ROM improvement between the SS and RT groups was not significantly different (*p* = 0.219, d = 0.24).

Table 3 shows the results for the passive torque at DF ROM and passive stiffness in both PRE and POST in each group. Passive torque at DF ROM showed a significant interaction effect. The *post hoc* test showed significant moderate and small magnitude increases after the intervention in the SS (*p* = 0.027, *d* = 0.64) and RT (*p* = 0.004, *d* = 0.41) groups, respectively, with no significant change in the control group (*p* = 1.000, *d* = −0.03). Again, the rate of change between the SS and RT groups showed no significant difference (*p* = 0.693, *d* = 0.17).

**TABLE 3:** Changes (mean ± SD) in passive torque at dorsiflexion range of motion (DF ROM and passive stiffness before (PRE) and after (POST) the intervention. The two-way repeated-measure ANOVA results (G × T: group × time interaction effect; F value), η_p_^2^, and Cohen’s d effect size are shown in the table below.

	SS		RT		Control		ANOVA results
	PRE	POST	PRE	POST	PRE	POST	*P* value, *F* value, η_p_^2^
Passive torque at DF ROM (Nm)	26.7 ± 6.8	32.0 ± 9.8*	31.0 ± 10.2	34.7 ± 8.2*	27.8 ± 8.0	27.5 ± 8.4	G x T: p = 0.028, F = 3.99 η_p_^2^ = 0.195
	d = 0.64		d = 0.41		d = −0.03		
Passive stiffness (Nm/°)	0.61 ± 0.16	0.56 ± 0.21*	0.71 ± 0.20	0.72 ± 0.20	0.68 ± 0.19	0.69 ± 0.19	G x T: p = 0.045, F = 3.41 η_p_^2^ = 0.171
	d = −0.32		d = 0.09		d = 0.02		

*Significant (*p* < 0.05) difference from the PRE, value where there are significant interaction effects.

Passive stiffness showed a significant interaction effect. The *post hoc* test showed a significant, small magnitude decrease after the intervention in the SS (*p* = 0.025, *d* = −0.32), with no significant (trivial magnitudes) change in the RT (*p* = 1.000, *d* = 0.09) and control group (*p* = 1.000, *d* = 0.02).

### MVC-ISO, MVC-CON, and MVC-ECC torque

Table 4 shows the results for the MVC-ISO, MVC-CON, and MVC-ECC torque in each group. MVC-ISO torque showed a significant interaction effect at 30° plantar flexion, neutral position, and 15° dorsiflexion. The *post hoc* test showed significant, large to moderate magnitude increases after the intervention in the RT group at 30° plantarflexion (*p* = 0.003, *d* = 1.00), neutral position (*p* = 0.008, *d* = 0.43), or 15° dorsiflexion (*p* = 0.007, *d* = 0.43), respectively. But, there were no significant changes (all were trivial magnitudes) in the SS and control group at 30° plantarflexion (SS: *p* = 1.000, *d* = 0.14; control: *p* = 1.000, *d* = 0.07), neutral position (SS: *p* = 1.000, *d* = 0.01; control: *p* = 1.000, *d* = 0.00), or 15° dorsiflexion (SS: *p* = 1.000, *d* = 0.04; control: *p* = 1.000, *d* = −0.06).

**TABLE 4:** Changes (mean ± SD) in the maximal voluntary isometric contraction (MVC-ISO) at different positions, maximal voluntary concentric contraction (MVC-CON) at 30°/s and 120°/s, and maximal voluntary eccentric contraction (MVC-ECC) at 30°/s before (PRE) and after (POST) the intervention. The two-way repeated-measure ANOVA results (G × T: group × time interaction effect; F value), η_p_^2^, and Cohen’s d effect size are shown in the table below.

	SS		RT		Control		ANOVA results
	PRE	POST	PRE	POST	PRE	POST	*P* value, *F* value, η_p_^2^
MVC-ISO at 30° plantarflexion (Nm)	48.9 ± 17.3	51.2 ± 15.0	49.6 ± 11.4	60.8 ± 11.2*	47.3 ± 20.5	48.9 ± 21.3	G x T: p = 0.012, F = 5.12 η_p_^2^ = 0.237
	d = 0.14		d = 1.00		d = 0.07		
MVC-ISO at neutral position (Nm)	136.6 ± 38.5	137.1 ± 31.6	130.3 ± 34.9	145.0 ± 32.5*	118.4 ± 47.4	118.6 ± 51.8	G x T: p = 0.038, F = 3.62 η_p_^2^ = 0.18
	d = 0.01		d = 0.43		d = 0.00		
MVC-ISO at 15° dorsiflexion (Nm)	180.7 ± 53.2	182.4 ± 40.1	163.6 ± 45.0	182.0 ± 41.9*	153.0 ± 63.3	149.0 ± 66.6	G x T: p = 0.034, F = 3.75 η_p_^2^ = 0.185
	d = 0.04		d = 0.43		d = −0.06		
MVC-CON at 30°/s (Nm)	117.9 ± 21.0	111.8 ± 16.5	112.2 ± 18.9	119.5 ± 20.1*	97.9 ± 33.5	99.1 ± 36.2	G x T: p = 0.039, F = 3.58 η_p_^2^ = 0.178
	d = −0.33		d = 0.38		d = 0.03		
MVC-CON at 120°/s (Nm)	70.7 ± 12.7	70.2 ± 11.0	69.7 ± 14.5	77.2 ± 13.1	62.4 ± 21.0	61.4 ± 19.2	G x T: p = 0.124, F = 2.23 η_p_^2^ = 0.119
	d = −0.04		d = 0.54		d = −0.05		
MVC-ECC at 30°/s (Nm)	182.5 ± 44.2	172.1 ± 30.1	171.6 ± 42.6	191.9 ± 41.6*	159.4 ± 64.7	164.6 ± 70.3	G x T: p = 0.026, F = 4.07 η_p_^2^ = 0.198
	d = −0.28		d = 0.48		d = 0.08		

*Significant (*p* < 0.05) difference from the PRE, value where there are significant interaction effects.

MVC-CON torque showed a significant interaction effect at 30°/s, but no significant interaction effect at 120°/s. In addition, with MVC-CON torque at 120°/s, there was no significant main effect for time (*p* = 0.286, *F* = 1.18, η_p_^2^ = 0.034). The *post hoc* test of MVC-CON torque at 30°/s showed a significant, moderate magnitude increase after the intervention in the RT (*p* = 0.009, *d* = 0.38) group, with no significant change in the SS (*p* = 0.297, *d* = −0.33) and control group (*p* = 1.000, *d* = 0.03).

MVC-ECC torque showed a significant interaction effect. The *post hoc* test showed a significant, moderate magnitude increase after the intervention in the RT (*p* = 0.023, *d* = 0.48) group, with no significant change in the SS (*p* = 0.767, *d* = −0.28) and control group (*p* = 1.000, *d* = 0.08).

### Muscle thickness and pennation angle

Table 5 shows the results for the muscle thickness and pennation angle in each group. Muscle thickness showed a significant interaction effect at MG and LG but no significant interaction effect at soleus. In addition, muscle thickness at the soleus showed no significant main effect for time (*p* = 0.057, *F* = 3.90, η_p_^2^ = 0.106). The *post hoc* test showed significant, moderate magnitude increases after the intervention in the RT group at MG (*p* = 0.002, *d* = 0.56) and LG (*p* = 0.001, *d* = 0.66). But, there were no significant changes in the SS and control group at MG (SS: *p* = 1.000, *d* = −0.05; control: *p* = 0.474, *d* = −0.11) or LG (SS: *p* = 1.000, *d* = -0.04; control: *p* = 0.736, *d* = −0.16).

**TABLE 5:** Changes (mean ± SD) in the muscle thickness at medial gastrocnemius (MG), lateral gastrocnemius (LG), and soleus and pennation angle at MG and LG before (PRE) and after (POST) the intervention. The two-way repeated-measure ANOVA results (G × T: group × time interaction effect; F value), η_p_^2^, and Cohen’s d effect size are shown in the table below.

	SS		RT		Control		ANOVA results
	PRE	POST	PRE	POST	PRE	POST	*P* value, *F* value, η_p_^2^
Muscle thickness at MG (mm)	18.7 ± 2.8	18.6 ± 3.1	17.9 ± 1.6	18.7 ± 1.5*	17.3 ± 2.9	17.0 ± 3.1	G x T: p < 0.001, F = 9.45 η_p_^2^ = 0.364
	d = −0.05		d = 0.56		d = −0.11		
Muscle thickness at LG (mm)	15.1 ± 1.8	15.0 ± 2.1	13.4 ± 2.1	14.9 ± 2.3*	14.2 ± 1.8	13.9 ± 2.2	G x T: p < 0.001, F = 10.15 η_p_^2^ = 0.378
	d = −0.04		d = 0.66		d = −0.16		
Muscle thickness at soleus (mm)	16.9 ± 2.0	17.1 ± 2.4	17.1 ± 2.8	17.8 ± 2.5	16.8 ± 2.1	16.9 ± 2.0	G x T: p = 0.36, F = 1.05 η_p_^2^ = 0.06
	d = 0.09		d = 0.25		d = 0.06		
Pennation angle at MG (°)	20.7 ± 3.3	21.1 ± 2.8	21.3 ± 3.8	23.4 ± 4.3	21.8 ± 5.7	21.7 ± 6.3	G x T: p = 0.106, F = 2.40 η_p_^2^ = 0.127
	d = 0.13		d = 0.56		d = −0.01		
Pennation angle at LG (°)	13.4 ± 2.0	14.0 ± 2.1	12.6 ± 2.1	13.3 ± 2.1	13.6 ± 2.1	13.9 ± 2.0	G x T: p = 0.75, F = 0.37 η_p_^2^ = 0.017
	d = 0.28		d = 0.33		d = 0.11		

*Significant (*p* < 0.05) difference from the PRE, value where there are significant interaction effects.

Pennation angle shows no significant interaction effect at MG and LG, with no significant main effect for time at MG (*p* = 0.071, *F* = 3.49, η_p_^2^ = 0.096) and LG (*p* = 0.071, *F* = 3.48, η_p_^2^ = 0.095).

### The relationship between change in DF ROM and passive torque at DF ROM, and also passive stiffness

**FIGURE 4:**
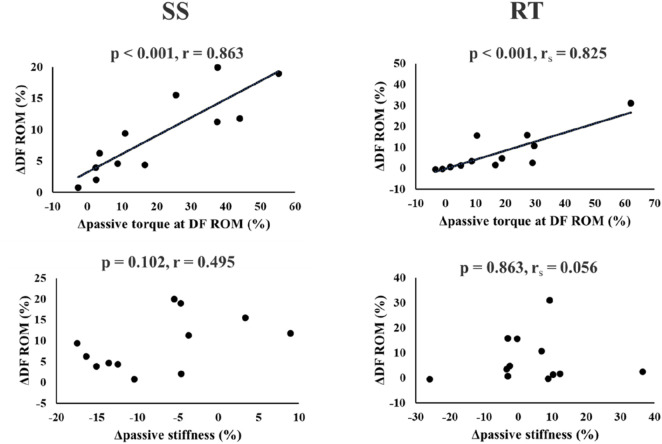
Relationship between change in dorsiflexion range of motion (DF ROM) and change in passive torque at DF ROM or passive stiffness in the SS and RT groups.

Significant positive correlations were found between DF ROM change and passive torque at DF ROM change in the SS (*p* < 0.001, *r* = 0.863) and RT (*p* < 0.001, *r*_*s*_ = 0.825) groups. But, no significant correlation was found between DF ROM change and passive stiffness change in the SS (*p* = 0.102, *r* = 0.495) and RT (*p* = 0.863, *r*_*s*_ = 0.056) groups (Figure 4).

## Discussion

In this study, we compared the effects of 6 weeks SS and RT on muscle passive properties, strength, muscle architecture, and the factors related to increased ROM. As hypothesized, DF ROM in both the SS and RT groups increased significantly, with no significant differences. On the other hand, passive stiffness was significantly decreased only in the SS group. MVC-ISO torque at 30° plantar flexion, neutral position, and 15° dorsiflexion, MVC-CON torque at 30°/s, and MVC-ECC torque at 30°/s MVC-ISO, MVC-CON, and MVC-ECC torque, and MG and LG muscle thickness increased only in the RT group, and the increase was greater in the RT group than in the control group. No interaction effects were found for MVC-CON torque at 120°/s, muscle thickness at the soleus, and pennation angle at MG and LG. In addition, there were significant positive correlations between DF ROM change and passive torque at DF ROM in both SS and RT groups, but no significant correlation between DF ROM change and passive stiffness in both groups.

A systematic review and meta-analysis reported similar increases in ROM with SS and RT (Alizadeh et al., 2023; Afonso et al., 2021). Our results showed a significant moderate- and small-magnitude increases after the intervention in the SS (p = 0.001, d = 0.65) and RT (p = 0.038, d = 0.37) groups, respectively. Alizadeh et al.'s systematic review with meta-analysis reported that RT significantly increased ROM with a moderate magnitude (effect size = 0.73) (Alizadeh et al., 2023). Interestingly, their subgroup analysis reported no significant ROM increase with RT using body weight exercises. Conversely, a systematic review on the chronic effects of RT with eccentric contraction demonstrated a large increase in lower-limb ROM (effect size = 0.86). Therefore, in this study, although RT involved body weight, the emphasis on eccentric contractions could produce a significant ROM increase. Thus, it is considered that the increase in DF ROM in the RT group was comparable to that in the SS group. In addition, it has been reported that the chronic effect of SS reduces the passive stiffness of MTU (Nakamura et al., 2012) and muscle stiffness (Takeuchi et al., 2023) but does not change muscle stiffness after a 6 weeks RT program (Ak et al., 2016). Therefore, our results support these previous studies regarding the effects of changes in ROM and passive stiffness in the present study. Since muscles possess viscoelasticity (Liesegang, 1990), prolonged stretching of the muscle by SS may cause a decrease in viscosity (thixotropic effects). The RT may not have decreased passive stiffness because the muscle was in the extended position for a shorter period of time than the SS, and thus did not significantly affect viscosity. In addition, Nakamura et al. (Nakamura et al., 2021b) reported that 4 weeks of SS increased ROM and decreased muscle stiffness. Still, no significant correlation was found between changes in ROM and changes in muscle stiffness, whereas a significant positive correlation was found between changes in ROM and changes in passive torque at DF ROM. Similarly, in this study, a significant positive correlation was found between the change in DF ROM and the change in passive torque at DF ROM in both SS and RT groups, but no significant correlation was found between the change in DF ROM and the change in passive stiffness. Since passive torque at maximal ROM is a measure of stretch tolerance (Weppler and Magnusson, 2010; Mizuno et al., 2013), the increase in ROM with SS and RT interventions might be due to changes in stretch tolerance. These results extend previous findings and strengthen the evidence that changes in stretch tolerance, are associated with increased ROM in SS and RT. In a previous study comparing the effects of SS at different intensities, it was reported that SS at higher intensities increased ROM and passive torque at DF ROM more than SS at lower intensities (Nakamura et al., 2021a). Therefore, the greater the mechanical stress applied to the MTU, the greater the effect of increased stretch tolerance should be. Since this study did not elucidate the mechanism of this change in stretch tolerance, it is necessary to investigate further whether there is a difference in the mechanism of the change in stretch tolerance between SS and RT interventions.

In the results of this study, the reason why muscle strength and thickness did not increase in the SS group may be related to the duration of SS. Sato et al. (2020a) investigated the chronic effect of SS training program at 360 s/week for 6-weeks for plantar flexors, and Nakamura et al. (2021a) investigated the chronic effect of SS training program at 540 s/week for 4 weeks, and there were no increases in muscle strength or muscle thickness. However, in previous studies by Warneke et al. (2023a), Warneke et al. (2022), who reported an increase in muscle strength and thickness with SS, SS was performed for 1 h daily for 6 weeks. In addition, (Yahata et al., 2021) performed SS for 1 h per week for 5 weeks at the maximum angle allowed on a stretch board and found no change in muscle thickness but significant increases in muscle strength. These results suggest that SS must have a prolonged duration to increase muscle strength and thickness. In animal models, passive stretching activates insulin-like and myogenic growth factors, stretch-activated channels, and the AKT/mTOR pathway and protein synthesis, which are important factors in muscle hypertrophy (Mohamad et al., 2011; Riley and Van Dyke, 2012; Tatsumi, 2010). In addition, Warneke et al.'s narrative review concludes that it is important to use high-volume stretching durations to increase maximal muscle strength and muscle mass with passive stretching in humans and animals (Warneke et al., 2023b). In the present study, SS time was 3 × 40 s thrice per week for 6 weeks (total 2,160 s), significantly less than previous studies (Warneke et al., 2023a; Yahata et al., 2021; Warneke et al., 2022) that found increases in muscle strength and thickness. Hence, the shorter volume or duration of stretching may be the rationale for the lack of increases in muscle strength and muscle thickness. However, further investigation is needed to explore the relationship between SS duration and increased muscle strength. For instance, Nelson et al. found that muscle strength gains were observed in students who were either physically inactive or minimally recreationally active, after 4 × 30 s of SS thrice per week for 10 weeks (Nakamura et al., 2025).

Namsawang et al. (2024) and Nakamura et al. (2025) reported that calf raise exercise with body weight also increased muscle strength, and in the present study, muscle strength measures other than MVC-CON torque at 120°/s were significantly increased in the RT group. In this study, muscle strength showed significant, large to moderate magnitude increases after the intervention in the RT group. Nakamura et al. (2025) investigated the effects of 8 weeks of eccentric contraction training emphasizing calf raise exercise in young, sedentary, healthy men. They reported a significant, large magnitude increase in MVC-ISO (effect size = 0.98). While this study used a 6 weeks program with 3 sessions per week (10 repetitions × 3 sets), the previous study employed an 8 weeks program with 2 sessions per week and progressively increasing set numbers. The difference in muscle strength gains due to variations in intervention duration and training protocols is an interesting point that warrants further study. Also, no significant interaction effect was observed in MVC-CON torque at 120°/s, and no change was observed in the RT group. One factor may be related to the principle of specificity in training (Hoffman, 2014; Behm and Sale, 1993) regarding the speed of movement. The RT in this study was performed with full ROM and 1-s concentric followed by a 3-s eccentric contraction, which is a slow movement speed, especially in the eccentric phase. Therefore, it is possible that the RT group showed a significant increase in MVC-CON torque at 30°/s and MVC-ECC torque at 30°/s but no significant change in MVC-CON torque at 120°/s, which has a higher speed of motion, even for the same dynamic contraction.

Although MVC torque and muscle thickness did not increase in the SS group in this study, it has been reported that SS performed for a longer duration and at high intensity can increase muscle strength and muscle thickness to a similar extent as RT (Warneke et al., 2023a; Warneke et al., 2022). Moreover, the narrative review by Behm et al. (2023) also presents the possibility that SS may be an alternative to RT to increase muscle strength and muscle hypertrophy in those who have the time but are not enthusiastic about moderate- or high-intensity RT. In the future, it will be necessary to consider appropriate intervention methods for each of these characteristics.

There are several limitations to this study. First, this study included healthy adult males with no lower extremity RT and SS habits. Therefore, it is unclear whether similar results would be obtained in subjects with different physical attributes, such as those with RT or SS habits, women, or older adults. Future studies should be conducted with different subjects. Second, the SS intensity performed in this study was defined as the maximum intensity without pain or discomfort. However, since a previous study reported that there was no significant correlation between stretch pain and passive peak torque during SS (Lim and Park, 2017), it is possible that this subjective SS intensity was not sufficiently quantified. Quantifying the SS strength using a load cell would be interesting, referring to the previous study (Warneke et al., 2023a). Third, the RT used in this study was the calf raise exercise performed with body mass. Therefore, exercise intensity may have varied among participants. It is also possible that there was a lack of training intensity progression as the exercise intensity remained constant during the intervention period, resulting in a lower exercise intensity in the second half of the intervention. In the future, it is necessary to standardize the load intensity among participants by using weights, bands, or other devices and to increase the load incrementally. Fourth, this study used ultrasound-measured muscle thickness as an index of muscle hypertrophy. Therefore, it is necessary to investigate the use of magnetic resonance imaging, the gold standard for measuring muscle mass, in the future. Fifth, researchers responsible for randomization and outcome assessments were aware of the study objectives, making complete blinding impossible. Nevertheless, standardized procedures and objective measurement tools were employed to minimize potential bias. Lastly, we compared RT and SS in plantar flexors in this study. To generalize these findings, future studies investigating other muscles, such as the hamstrings and quadriceps, will be necessary.

In clinical application, our results suggest that RT performed through a full ROM could increase ROM to a similar extent as SS while simultaneously increasing muscle strength and thickness. For athletes and coaches, RT could serve as a time-efficient strategy to achieve both flexibility and strength gains within a single program, potentially reducing the need for SS sessions. Increased muscle stiffness is one of the risk factors for sports injuries (Watsford et al., 2010; Pickering Rodriguez et al., 2017), and SS could prevent muscle injuries (Takeuchi et al., 2024). Thus, if a decrease in passive stiffness is the goal, SS should be selected instead of RT. Furthermore, RT can be expected to increase ROM while also promoting strength gains and muscle hypertrophy. The information from the current study indicates that clinicians and athletes should select RT or SS based on their specific goals.

## Conclusion

This study compared plantar flexors’ passive and active properties between 6-weeks of SS and RT and a control group without intervention. As a result, SS and RT caused similar ROM increases. However, passive stiffness decreased only in SS, and most muscle strength and thickness measures increased only in RT. When implementing SS and RT, it is necessary to understand the differences in the effects obtained from SS and RT, and to select SS and RT according to the objectives.

## Data Availability

The raw data supporting the conclusions of this article will be made available by the authors, without undue reservation.
